# A Novel Fluorescence-Based Screen of Gene Editing Molecules for Junctional Epidermolysis Bullosa

**DOI:** 10.3390/ijms24065197

**Published:** 2023-03-08

**Authors:** Janine Zwicklhuber, Thomas Kocher, Bernadette Liemberger, Stefan Hainzl, Johannes Bischof, Dirk Strunk, Anna M. Raninger, Iris Gratz, Verena Wally, Christina Guttmann-Gruber, Josefina Piñón Hofbauer, Johann W. Bauer, Ulrich Koller

**Affiliations:** 1 EB House Austria, Research Program for Molecular Therapy of Genodermatoses, Department of Dermatology and Allergology, University Hospital of the Paracelsus Medical University, 5020 Salzburg, Austria; 2 Cell Therapy Institute, Spinal Cord Injury and Tissue Regeneration Center Salzburg (SCI-TReCS), Paracelsus Medical University, 5020 Salzburg, Austria; 3 Department of Biosciences and Medical Biology, University of Salzburg, 5020 Salzburg, Austria; 4 Department of Dermatology and Allergology, University Hospital of the Paracelsus Medical University, 5020 Salzburg, Austria

**Keywords:** *COL17A1*, junctional epidermolysis bullosa, collagen type XVII (C17), green fluorescent protein (GFP), CRISPR molecule screening

## Abstract

Junctional epidermolysis bullosa (JEB) is a severe blistering skin disease caused by mutations in genes encoding structural proteins essential for skin integrity. In this study, we developed a cell line suitable for gene expression studies of the JEB-associated *COL17A1* encoding type XVII collagen (C17), a transmembrane protein involved in connecting basal keratinocytes to the underlying dermis of the skin. Using the CRISPR/Cas9 system of *Streptococcus pyogenes* we fused the coding sequence of GFP to *COL17A1* leading to the constitutive expression of GFP-C17 fusion proteins under the control of the endogenous promoter in human wild-type and JEB keratinocytes. We confirmed the accurate full-length expression and localization of GFP-C17 to the plasma membrane via fluorescence microscopy and Western blot analysis. As expected, the expression of GFP-C17^mut^ fusion proteins in JEB keratinocytes generated no specific GFP signal. However, the CRISPR/Cas9-mediated repair of a JEB-associated frameshift mutation in GFP-*COL17A1*^mut^-expressing JEB cells led to the restoration of GFP-C17, apparent in the full-length expression of the fusion protein, its accurate localization within the plasma membrane of keratinocyte monolayers as well as within the basement membrane zone of 3D-skin equivalents. Thus, this fluorescence-based JEB cell line provides the potential to serve as a platform to screen for personalized gene editing molecules and applications in vitro and in appropriate animal models in vivo.

## 1. Introduction

The inherited blistering skin disease epidermolysis bullosa is associated with severe debilitating conditions, which significantly impact patients’ quality of life [1]. In general, the severity and heterogeneity of the disease justifies the ongoing development of therapeutic options at the DNA and RNA level, of which some are at the preclinical development stage, whereas others have already been applied clinically [2]. Furthermore, cDNA replacement and splicing-modulating therapies, gene editing via designer nucleases, in particular, has become the main focus of research over the last years. Although clinical translation remains a big challenge, designer nucleases such as CRISPR/Cas9 are constantly improving in regards to efficiency and safety and therefore hold great promise for future clinical applications as therapeutics for genodermatoses [3,4,5]. For the junctional form of EB (JEB), mutations in genes encoding integrin-α6β4, laminin-332, and type XVII collagen (C17) are the predominant cause of disease [6]. We have recently developed patient-specific Cas9 nuclease and nickase-based targeting strategies to either reframe [7] or repair [8] the *COL17A1* gene using endogenous end joining (EJ) as well as homology-directed repair (HDR) pathways in JEB keratinocytes. Mutations in *COL17A1* result in reduced or absent expression of type XVII collagen (C17), a transmembrane protein that is necessary for the connection of the basal keratinocytes of the epidermis to the underlying lamina lucida [9,10]. Using a *COL17A1* reframing approach, we could successfully restore C17 expression in 46% of treated primary JEB keratinocytes. The reframed C17 protein variants expressed, encoded *COL17A1* transcripts predominantly carrying 25- and 37-nt deletions [7]. Additional inclusion of a single-stranded oligonucleotide (ssODN) template for HDR increased C17 restoration levels up to 60% as assessed by flow cytometry [8]. Here, the combined induction of EJ and HDR led to a significant boost of gene repair/reframing and, most importantly, to an increased adhesive strength of C17 to its binding partner laminin-332, as well as an accurate deposition of C17 along the basement membrane zone (BMZ) of 3D-skin equivalents upon epidermal stratification. However, as the downstream analyses of *COL17A1* editing outcomes are time-consuming and costly, we developed a screening system to facilitate and accelerate the detection of C17 restoration, based on the incorporation of the GFP reporter cassette directly upstream of the coding sequence for C17 in both wild-type (WT) and JEB human keratinocytes, keeping the expression of the chimeric gene under the control of the endogenous *COL17A1* promotor.

## 2. Results

### 2.1. GFP-C17 Expression upon Stable CRISPR/Cas9-Mediated GFP Integration

**Figure 1:**
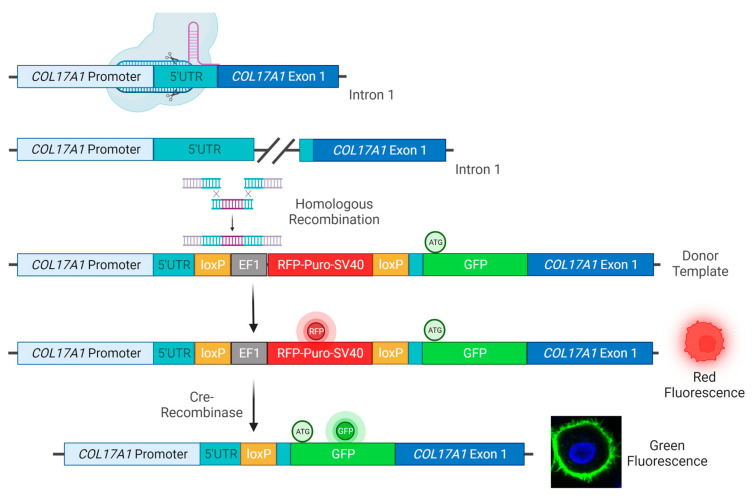
CRISPR/Cas9-mediated integration of GFP at the COL17A1 locus. Upon delivery of a Cas9/sgRNA-expressing plasmid and an HR template, comprising an RFP-puromycin selection cassette and GFP flanked by a 5′ and 3′ HA for homologous recombination, into human WT and JEB keratinocytes the Cas9 nuclease/nickase performs a double/single-strand DNA break within the 5′UTR of COL17A1. This DNA damage induces HDR at the COL17A1 locus leading to the integration of the selection cassette and GFP at the target site. The RFP-puromycin selection cassette, initially required for the isolation of cell clones in which the HR template sequence is integrated, was then removed via Cre-recombinase-treatment leaving one loxP site as a DNA footprint within the UTR. The GFP reporter molecule was then fused in frame to the COL17A1 gene via a short Gly-Ser-linker sequence leading to the expression of a GFP-C17 fusion protein in the cell under expression control of the endogenous promoter. A pure GFP^pos^/RFP^neg^ cell population was then obtained via FACS. Created with BioRender.com.

In order to fuse GFP N-terminally to C17, WT, and JEB keratinocytes were co-transfected with a sgRNA/Cas9-expressing-plasmid targeting the 5′UTR of *COL17A1* and a donor plasmid for homologous recombination (HR). We used an E6/E7-immortalized JEB keratinocyte line harboring a homozygous frameshift mutation (c.3899-3900delCT/c.3899-3900delCT) within exon 52 of *COL17A1*. This cell line was previously shown to be negative for full-length C17 expression [7]. The HR template contained a floxed RFP-puromycin selection cassette, as well as the full-length sequence of GFP flanked by two *COL17A1* homology arms (5′HA and 3′HA) for HDR induction. The start codon in exon 1 of *COL17A1* within the 3′HA was deleted (Figure 1).

**Figure 2:**
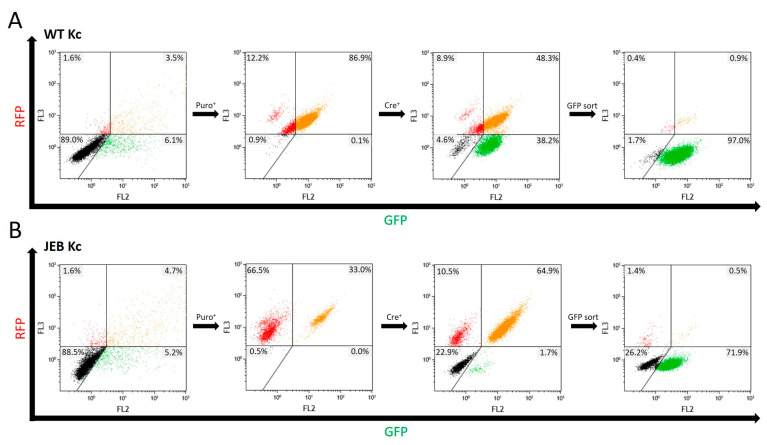
Flow cytometric analyses during GFP-C17 cell line generation. Following treatment of wild-type keratinocytes (**A**) and JEB keratinocytes (**B**), transfected cells transiently expressed RFP and GFP from the HR template and the Cas9/sgRNA-expressing plasmid, respectively. Subsequent puromycin selection led to the accumulation of RFP- or RFP/GFP-expressing cells. Cre-recombinase-mediated deletion of the selection cassette brings the GFP-C17 expression under the control of the endogenous COL17A1 promoter resulting in a cell population exclusively emitting green fluorescence (~38% of WT Kc and ~1.7% of JEB Kc). A subsequent GFP^pos^ sort via FACS significantly increased the purity of this cell fraction. A total of ~97% of treated WT Kc and ~72% of treated JEB Kc solely displayed a green signal following FACS sorting and expansion.

Flow cytometric analysis was performed after each treatment step to monitor the success of the cell line generation. Upon co-transfection with the Cas9/sgRNA-expressing plasmid and the HR template plasmid, both WT and JEB keratinocytes expressed RFP-Puro and GFP from the HR template and the Cas9/sgRNA-expressing plasmid, respectively (Figure 2). Keratinocytes with successfully integrated HR templates were then selected via puromycin treatment. A subsequent Cre-recombinase-mediated deletion of the selection cassette brought the GFP-C17 expression under the control of the endogenous *COL17A1* promoter. This resulted in a cell population exclusively emitting green fluorescence (~38% of WT Kc and ~1.7% of JEB Kc). To further purify the GFP-C17-expressing cell population, a GFP^pos^/RFP^neg^ sort was implemented, resulting in ~97% of treated WT Kc and ~72% of treated JEB Kc displaying a green signal detectable by flow cytometric analysis (Figure 2). Notably, the median fluorescence intensities (MFI) of GFP varied between both keratinocyte populations. As expected, the GFP signal is higher in GFP-sorted WT keratinocytes (MFI: 5255) due to normal expression of GFP-C17 in the cells. In comparison, in GFP-sorted JEB keratinocytes, in which C17 expression is disrupted, the intensity of the GFP signal is lower (MFI: 2681). In this case, sorting for GFP-positive cells enriched for those cells with residual levels of truncated GFP-C17^mut^ proteins, as the homozygous frameshift mutation in exon 52 results in a premature termination codon.

### 2.2. GFP-C17 Fusion Protein Is Correctly Expressed at the Plasma Membrane

**Figure 3:**
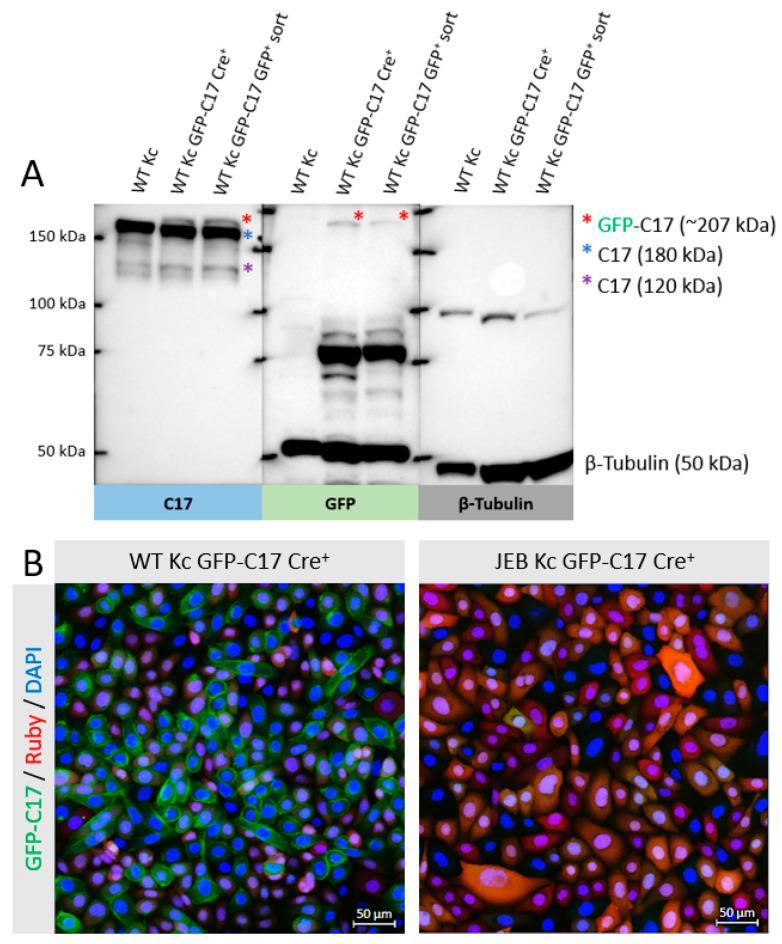
GFP-C17 expression and localization in WT and JEB keratinocytes. (**A**) Western blot analysis of cell lysates of WT Kc GFP-C17 revealed the expression of the full-length GFP-C17 fusion protein detectable upon GFP and C17 staining above the band corresponding to wild-type C17 (180 kDa). The shed extracellular C17 domain has a molecular weight of 120 kDa. Lysates from WT keratinocytes without GFP fusion served as a negative control. β-tubulin (50 kDa) was used as a loading control. (**B**) Fluorescence microscopic analysis of WT Kc expressing GFP-C17 after Cre-recombinase-treatment prior to GFP^pos^ selection via FACS revealed the accurate localization of the fusion protein within the plasma membrane. As expected, JEB cells expressing GFP-C17^mut^ emitted no GFP signal. Cells were stained with 4′, 6-Diamidino-2-phenylindol (DAPI) for cell nuclei visualization.

To evaluate and quantify GFP-C17 fusion protein levels, Western blot analysis of cell lysates from WT Kc expressing GFP-C17 was performed. The full-length GFP-C17 fusion protein (~207 kDa) was detected above the band corresponding to wild-type C17 (180 kDa) upon GFP and C17 staining (Figure 3A). Furthermore, the 120 kDa extracellular domain of C17, which is constitutively shed from the cell surface in cultured keratinocytes [11,12], was also detectable in edited WT Kc using the C17-specific antibody, whereas a GFP-containing protein of >75 kDa could be detected in the GFP blot that potentially represents the GFP-tagged N-terminal domain (predicted size ~87 kDa) that remains following cleavage of the ectodomain. To further elucidate the expression and localization of the GFP-C17 fusion protein, confluent monolayers of WT Kc and JEB Kc expressing GFP-C17/GFP-C17^mut^ after Cre-recombinase-treatment and before GFP^pos^ selection were subjected to fluorescence microscopic analysis. WT Kc revealed an accurate expression and localization of the fusion protein within the plasma membrane (Figure 3B). In contrast, JEB cells expressing GFP-C17^mut^ displayed no GFP-C17. However, this outcome was expected, as JEB keratinocytes do not express full-length C17, and an accurate expression of full-length GFP-C17 fusion protein was therefore not presumed. Furthermore, PCR analysis on the genomic DNA of WT and JEB Kc expressing GFP-C17 confirmed the accurate genomic integration of GFP at the *COL17A1* locus (Figure S1A).

**Figure 4:**
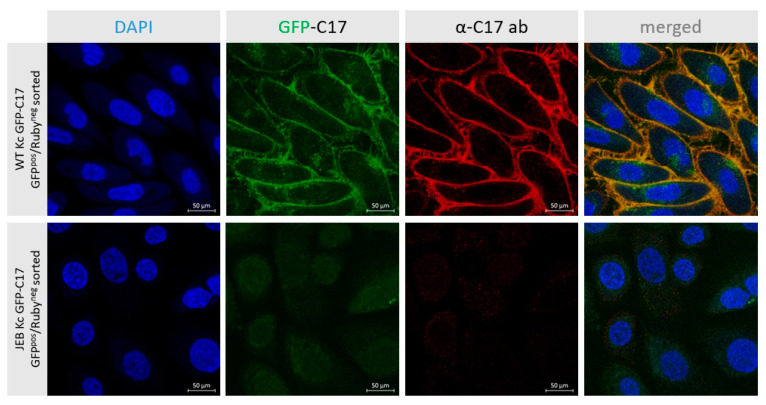
GFP-C17 expression and localization in WT and JEB keratinocytes upon GFP^pos^/RFP^neg^ sorting. Fluorescence (GFP) and immunofluorescence (C17) microscopy revealed the accurate deposition of the GFP-C17 fusion protein within the plasma membrane of GFP-C17-sorted WT Kc, whereas in sorted JEB Kc the GFP-C17^mut^ expression was hardly detectable within the cytoplasm. Merging of both fluorescence channels revealed a clear overlap in the expression of plasma membrane-integrated immunolabelled C17 (C17 and GFP-C17) and GFP-C17 proteins directly emitting green fluorescence. Nuclei were stained with 4′, 6-Diamidino-2-phenylindol (DAPI, blue).

To further evaluate the correct localization of GFP-C17 fusion protein, GFP^pos^/RFP^neg^ sorted WT and JEB keratinocytes were additionally stained with a C17-specific antibody. WT Kc expressing GFP-C17 displayed a fluorescence signal for both, C17 (red) and GFP-C17 fusion protein (green) in the plasma membrane (Figure 4). Merging the channels shows a complete overlap of C17 and GFP-C17 (orange), confirming the correct localization of the fusion protein in the plasma membrane. In contrast, JEB Kc expressing GFP-C17^mut^ showed no specific fluorescence signals.

### 2.3. COL17A1 Repair via CRISPR/Cas9 Restores GFP-C17 Expression in JEB Kc

**Figure 5:**
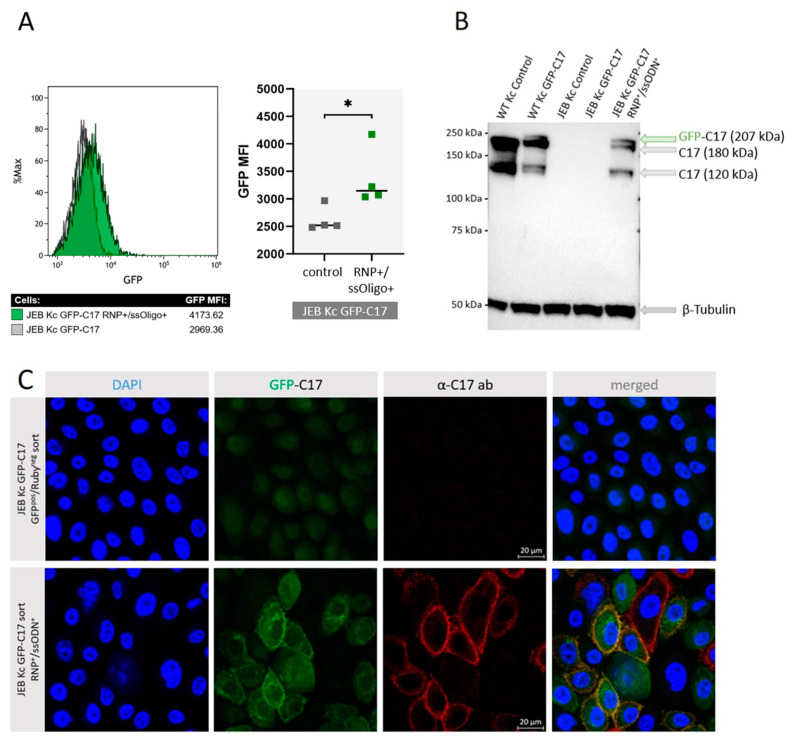
CRISPR/Cas9-mediated GFP-C17 restoration in JEB keratinocytes. (**A**) As a result of the RNP/ssODN-treatment of GFP-C17^mut^-expressing JEB cells flow cytometric analysis showed a significant (*) increase of the median GFP signal intensity in treated cells upon gene repair in four individual experiments. Statistical analyses (paired Student’s *t*-test) were performed using GraphPad Prism 9. (**B**) Western blot analysis of cell lysates from RNP/ssODN-treated GFP-C17^mut^-expressing JEB cells showed restored full-length C17 (180 kDa), full-length GFP-C17 (~207 kDa) as well as the presence of the C17 ectodomain at 120 kDa. (**C**) Immunofluorescence microscopy showed no fluorescence in untreated JEB GFP-C17^mut^-expressing JEB cells, whereas RNP/ssODN treatment led to the accurate restoration and integration of full-length GFP-C17 within the plasma membrane of edited cells. The cell’s nuclei were stained with 4′, 6-Diamidino-2-phenylindol (DAPI, blue).

To test the resulting JEB GFP-C17^mut^ cell line we treated the cells with a previously described RNP/ssODN combination for *COL17A1* repair [8]. Flow cytometry revealed a clear shift in the GFP fluorescence intensity of the cell population upon treatment. In four individual experiments, we achieved a significant increase in median GFP signal intensity in the *COL17A1*-edited JEB GFP-C17^mut^ cells versus untreated controls. Western blot analysis of cell lysates from RNP/ssODN-treated JEB Kc expressing GFP-C17 further confirmed the partial restoration of C17 expression. Notably, full-length GFP-C17 (~207 kDa), full-length C17 (180 kDa), and the C17 ectodomain (120 kDa) were detected in CRISPR-treated JEB keratinocytes (Figure 5B). Immunofluorescence analysis confirmed increased GFP fluorescence in corrected cells over the background expression in untreated cells. Moreover, GFP staining was present in the plasma membrane in a fraction of the CRISPR-edited cells, indicative of successful restoration of functional GFP-C17. Additional staining with the C17-specific antibody demonstrated membrane co-staining, thereby confirming accurate restoration and plasma membrane integration of full-length GFP-C17 fusion protein in a fraction of RNP/ssODN-treated cells (Figure 5C). Untreated GFP-C17^mut^ JEB Kc showed no fluorescence with the C17 antibody. Finally, the genomic integration of the donor template in RNP/ssODN-treated GFP-C17^mut^-expressing JEB cells was also confirmed via PCR (Figure S1B).

### 2.4. GFP-C17 Protein Is Deposited at the Correct Location within Organotypic 3D Cultures

**Figure 6:**
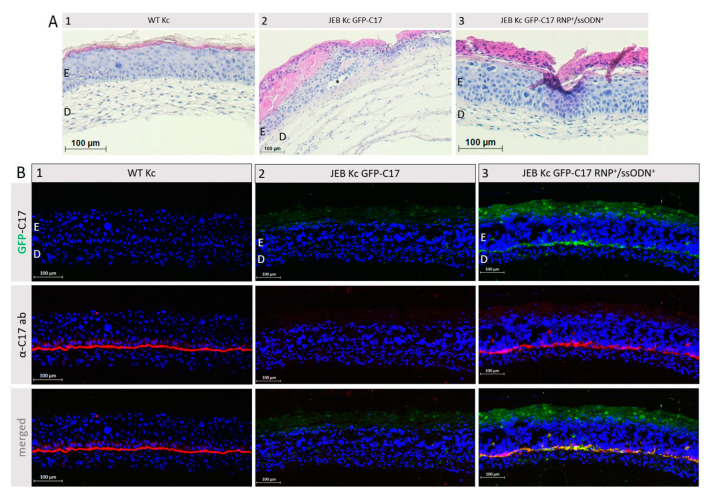
GFP-C17 restoration within the BMZ of JEB-derived skin equivalents. (**A**) H&E staining of skin equivalents, expanded from WT Kc (1) as well as untreated (2) and RNP/ssODN-treated (3) GFP-C17^mut^-expressing JEB cells, revealed a normal epidermal stratification, although in SEs derived from untreated GFP-C17^mut^-expressing JEB cells a blister (*) was detectable within the BMZ. E = Epidermis; D = Dermis. (**B**) Immunofluorescence staining performed on cryosections showed an accurate C17 expression (red fluorescence) in the BMZ of SEs derived from WT keratinocytes (1). SEs from untreated GFP-C17^mut^-expressing JEB Kc showed no visible GFP-C17 and C17 expression (2), whereas immunofluorescence staining of 3D SEs expanded from RNP/ssODN-treated (~11% GFP-C17^pos^) JEB cells revealed the accurate deposition of GFP-C17 (green fluorescence) within the BMZ overlapping with immunolabelled C17/GFP-C17 (3). The cell’s nuclei were stained with 4′, 6-Diamidino-2-phenylindol (DAPI, blue).

To further verify the correct localization of restored GFP-C17, immortalized WT fibroblasts and either WT Kc, untreated or RNP/ssODN-treated GFP-C17^mut^-expressing JEB Kc were used to generate full-thickness skin equivalents (SE). Haematoxylin and Eosin (H&E) staining confirmed the correct formation of the skin layers revealing a normal epidermal stratification (Figure 6A). In SEs derived from untreated GFP-C17^mut^-expressing JEB cells blister formation within the BMZ was observed. Using a C17-specific antibody, staining of cryosections from SEs expanded from RNP/ssODN-treated GFP-C17^mut^-expressing JEB cells revealed a clear GFP-C17 restoration similar to the C17 expression in SEs derived from WT Kc (Figure 6B). A homogenous GFP signal was detected along the BMZ, confirming the accurate deposition of GFP-C17 between the dermal and the epidermal layer. Furthermore, the GFP signal colocalized with the detection of C17 via antibody staining. Additionally, colocalization of restored GFP-C17 with its key binding partner laminin-332 in human skin was evident (Figure S3).

## 3. Discussion

In this study, we generated a reporter cell line that would be suitable for conducting protein-protein interaction studies or, for our purpose, the selection and comparison of gene editing strategies/molecules for junctional EB. The direct labeling of mutant C17 with GFP enables a fast screening procedure for *COL17A1*-specific gene editing molecules, as the repair outcome can be subsequently analyzed live upon treatment of the cells according to the respective fluorescence signal generated. Currently, in our JEB-screening cell line, we have a mixture of cells carrying GFP-*COL17A1* on one or both alleles. Subsequent CRISPR/Cas9-mediated C17 repair can lead to functional correction in none, one, or both alleles. Depending on which type of correction takes place on which allele, different staining patterns can be observed, resulting in heterogenous outcomes in bulk-treated cells as seen in our immunofluorescence analyses of JEB keratinocyte monolayers, as well as of 3D skin equivalents. Only edits at the GFP-*COL17A1* allele that restores the reading frame would result in enhanced reporter expression, whereas edits at the parental (non-GFP) allele that result in functional protein expression can only be detected by additional C17-antibody staining. As such, based on GFP expression alone, we would underestimate the functional correction efficiencies achieved by our gene editing strategies. In order to increase the robustness of our screening system, we are now planning to isolate and expand single-cell clones in which the GFP cassette is accurately fused to *COL17A1* in a homozygous manner. This will decrease the heterogeneity of the outcomes observed and enable us to directly distinguish and estimate total protein restoration (increased GFP expression) from functional protein restoration (membrane GFP expression), thereby further enhancing the utility of the cell line.

Once optimized the fluorescent JEB cell line can be used for protein restoration analysis upon gene editing in vivo using appropriate mouse models as recently described [13]. Currently, it is necessary to conduct time- and resource-intensive immunofluorescence stainings on fixed keratinocytes in order to evaluate the expression and localization of C17 within JEB Kc [7,8]. By using our novel GFP-C17 cell line we are now able to perform live cell imaging directly after treatment, thereby facilitating and accelerating the analysis of new gene editing platforms. This includes recently developed technologies such as prime editing, as well as future gene editing technologies [14].

## 4. Materials and Methods

### 4.1. Cell Culture & Cell Lines

JEB keratinocytes (JEB-282-Kc) were isolated from a patient biopsy and carry a homozygous frameshift mutation (c.3899-3900delCT/c.3899-3900delCT) within exon 52 of *COL17A1* [7,8]. Wild-type keratinocytes (hKc-1090 and WT-200-Kc) and wild-type fibroblasts (HC-941-Fibs) were isolated from healthy donors upon informed consent. All cells were subsequently immortalized through transduction of the human papillomavirus (HPV) proteins E6 and E7 and grown at 37 °C and 5% CO_2_ in a humidified incubator [15]. WT Kc as well as JEB Kc were cultured in CnT-Prime Epithelial Proliferation Medium (CELLnTEC, Bern, Switzerland) with primocin (InvivoGen, Toulouse, France). Wild-type fibroblasts were cultivated in CnT Fibroblast Medium (CELLnTEC, Bern, Switzerland) with primocin (InvivoGen, Toulouse, France).

### 4.2. Transfection and Electroporation of Keratinocytes and Fibroblasts

The sgRNA was amplified from the genomic DNA of WT Kc using the following primer combination: fw primer: 5′-ATCCGGTTATCAGCTTCAACAGTG-3′; rv primer: 5′-GGTTATCAGCTTCAACAGTGGTTT-3′. The PCR product was cloned as double-stranded DNA oligonucleotide into the CMV-hspCas9-T2A-GFP-H1-gRNA linearized SmartNuclease^TM^ and SmartNickase^TM^ vector (System Biosciences, Palo Alto, CA, USA) according to the manufacturer’s protocol.

The homology arms (HA) for homologous recombination were amplified from genomic DNA isolated from WT Kc using a forward primer (5′- GATCGAATTCGAATGTTTCATACTTTGAGAAGAGTAAAGTTCCATAC-3′) and a reverse primer (5′-GATCAGATCTTGTTGAAGCTGATAACCAAAAACATGATTTCAAGAATT-3′) specific for the 5′HA and a forward primer (5′-GATCGGATCCGGCGCAGGAGCCGGCGCCACCGATGTAACCAAGAAAAACAAACGAGATGGAAC-3′) and a reverse primer (5′-GATCGCATGCTGACTTAGAGTTGGTAGGAGGAGAGTTTAAATG-3′) specific for the 3′HA, respectively. The PCR products were cloned into HR Targeting Vector HR110PA-1 (MCS1-EF1α-RFP-T2A-Puro-pA-MCS2) (System Biosciences, Palo Alto, CA, USA), carrying an RFP-Puromycin selection cassette flanked by loxP sites. The GFP sequence was amplified from pMXs-IRES-GFP Retroviral Vector (Cell Biolabs, San Diego, CA, USA) using a GFP-specific forward (5′-GATCGGATCCGTGGGGACCTATAATTACTAAAAACAATGATAAGTATGACTATCATGGTTTCTGATTTTTCCTGCAGGTGGCTATGGTATGGTGAGCAAGGGCGAGGAGC-3′) and reverse (5′-GATCGGATCCGGCGCCTGCTCCCTTGTACAGCTCGTCCATGCC-3′) primer. The nucleotide sequence 5′-ggagcaggcgccGGATCCggcgcaggagccggcgccacc-3′ encoding the Gly-Ser-Linker for GFP-C17 fusion was included in the 3′HA forward and GFP reverse primer, respectively.

Keratinocytes were grown to a confluency of 80–90% in 6-well plates and transfected with 1.5 µg of each Cas9/sgRNA-expressing plasmids and HR donor plasmid and 0.3 µL Xfect™ Transfection Reagent per µg plasmid DNA according to the manufacturer’s protocol (Takara Bio, Kusatu, Japan).

For restoration of C17 in the JEB GFP-C17^mut^ screening cell line, the cells were trypsinized and washed with PBS prior to nucleofection using the Neon™ Transfection System 10 μL Kit (Invitrogen, Waltham, MA, USA). For RNP complexing, 0.3 µL of spCas9 (IDT, Coralville, IA, USA) and 0.486 µL of sgRNA (IDT, Coralville, IA, USA) were mixed and incubated for 10 min at RT. Then, 250 ng of the HR donor template was added to the RNPs. 3 × 10^5^ keratinocytes were resuspended in buffer “R” provided in the Neon Transfection Kit, mixed with pre-complexed RNPs, and electroporated using the Neon transfection system according to Petkovic et al. [8]. Treated cells were then seeded into antibiotic-free CnT-Prime Medium (CELLnTEC, Bern, Switzerland) in 6-well plates. After 1 day of incubation, primocin (InvivoGen, Toulouse, France) was added.

### 4.3. Flow Cytometric Analysis and FACS

Transfection efficiency was evaluated via flow cytometric analysis 48 h post-transfection using a Beckman Coulter FC500 FACS analyzer (Beckman Coulter, Brea, CA, USA). Data analysis was performed using the Kaluza software (Beckman Coulter, Brea, CA, USA). Statistical analysis was performed using GraphPad Prism 9 (GraphPad Software, La Jolla, CA, USA) for paired Student’s *t*-tests.

For Flow Cytometry mediated sorting of GFP^pos^ cells, cells were trypsinized and washed with PBS. They were maintained in PBS and sorted using the FACS Aria III cell sorter (BD Biosciences, Franklin Lakes, NJ, USA).

### 4.4. Immunofluorescence Staining of C17 in Keratinocytes

5 × 10^4^ cells were seeded into chamber slides and fixed with 4% Para-formaldehyde for 10 min at RT upon reaching confluency. After three washing steps with 1× PBS, cells were incubated with anti-collagen type XVII rabbit monoclonal antibody (#184996; Abcam, Cambridge, UK), diluted 1:1000 in 1× blocking reagent (Roche Diagnostics, Vienna, Austria) for 1 h at RT. Cells were incubated for 1 h with Alexa-Fluor-488 goat anti-rabbit or Alexa-Fluor-594 goat-anti-rabbit (Thermo Fisher Scientific, Waltham, MA, USA), diluted 1:400 in PBS, in combination with 4′, 6-Diamidino-2-phenylindol (DAPI) (1:2000) (VWR, Vienna, Austria). Cells were stored in PBS and analyzed using the confocal laser scanning microscope Axio Observer Z1 attached to LSM700 (Carl Zeiss, Jena, Germany).

### 4.5. DNA Isolation and Integration PCR

Genomic DNA of keratinocytes was isolated using the ReliaPrep™ Blood gDNA Miniprep System kit (Promega, Madison, WI, USA), according to the manufacturer’s protocol. Integration of the donor template was detected by PCR analysis using a specific forward primer (5′-GGGCGACACCCTGGTGAACC-3′) binding within the GFP sequence and reverse primer (5′- CCTGGGCAACAGAGCAAGACTCCGTCTC-‘3) binding Intron 2 of *COL17A1.* As well as with specific forward primer (5′-GGGACGGGACCCTTGCTGTGCATTCC-3′) binding Intron 1 of *COL17A1* and reverse primer (5′-GGTTCACCAGGGTGTCGCCC-3′) binding GFP.

### 4.6. Protein Isolation and Western Blot Analysis

Protein lysates were collected from cell pellets using radioimmunoprecipitation assay (RIPA) buffer (Santa Cruz Biotechnology, Heidelberg, Germany). For loading onto an 8% BisTris-Gel (Invitrogen, Waltham, MA, USA), cell lysates were mixed with a 4× loading buffer (0.25 M Tris, 8% SGS, 30% glycerol, 0.02% bromophenol blue [pH 6.8]) and denatured at 95 °C for 5 min. Western blot analysis was performed as previously described [7,16]. The nitrocellulose membrane was blocked with 10× blocking reagent from Roche Diagnostics (Roche Diagnostics GmbH, Mannheim, Germany) diluted 1:10 in Tris-buffered saline with 0.2% Tween (TBS-T) for 1 h at room temperature. C17 was detected via a monoclonal anti-C17 antibody (#184996) (Abcam, Cambridge, UK) at a dilution of 1:2000 in TBS-T. The membrane was incubated overnight at 4 °C with the primary antibody. GFP detection was performed using a GFP-specific antibody (MBL International Corporation, Woburn, MA, USA) diluted 1:300 in blocking reagent (Roche Diagnostics GmbH, Mannheim, Germany) (diluted 1:10 in TBS-T). Antibody staining against β-tubulin (ab6064; Abcam, Cambridge, UK) was used as loading control in a dilution of 1:2000. A goat anti-rabbit HRP-labelled antibody (Dako, Santa Clara, CA, USA) was used as a secondary antibody. The membrane was incubated with the secondary antibody for 1 h at a dilution of 1:300 in TBS-T. Protein band visualization was performed using the Immobilon Western Chemiluminescent HRP Substrate (Merck, Darmstadt, Germany) and the ChemiDoc XRS Imager (BioRad, Hercules, CA, USA).

### 4.7. Generation of Skin Equivalents

A human fibrin scaffold was used for the generation of skin equivalents. 1 × 10^5^ fibroblasts (HC-941-Fibs) were immersed in a fibrinogen scaffold consisting of DMEM with 20% FCS, fibrinogen (F4883; Sigma–Aldrich, St. Louis, MO, USA) in 0.9% NaCl (final concentration = 25 mg/mL), thrombin (T8885; Sigma–Aldrich, St. Louis, MO, USA) dissolved in 25 mM CaCl_2_ and aprotinin (A6279; Sigma–Aldrich, St. Louis, MO, USA). The scaffold was directly prepared in Falcon^®^ permeable support inserts with a 0.4 µm transparent PET membrane (Corning, New York, NY, USA) and placed in BioCoat™ Deep-Well Plates (6-well (Corning, New York, NY, USA) for 1 h at 37 °C and 5% CO_2_. 1 × 10^6^ keratinocytes (HC-941-Kc, JEB-282-Kc GFP-C17^mut^ or JEB-282-Kc GFP-C17^mut^ RNP/ssODN treated) per well were seeded on top of the matrix and grown to confluence in DMEM:Ham’s F-12 Green’s keratinocyte medium. Skin equivalents were then raised to an air-liquid interface and cultured for 28 days to allow stratification, before harvesting. SEs were either fixed in OCT (Scigen, Tissue-Plus, Paramount, CA, USA) for cryosectioning or placed into 4% PFA for subsequent paraffin embedding.

### 4.8. Immunofluorescence Staining of Skin Equivalents

Cryosections of 8 µm were fixed with acetone:methanol (1:1) at −20 °C for 15 min and washed twice with PBS for 5 min. The slides were then incubated for 1.5 h with a human-specific anti-C17 antibody (#184996; Abcam, Cambridge, United Kingdom) diluted 1:500 in 1× blocking reagent (Roche Diagnostics GmbH, Mannheim, Germany) in TBS-T. After three washing steps with PBS, secondary antibody Alexa Fluor 549 goat anti-rabbit IgG (H + L) (Thermo Fisher Scientific, Waltham, MA, USA) diluted 1:500 in PBS and DAPI (4′,6-Diamidino-2-phenylindol) (Thermo Fisher Scientific, Waltham, MA, USA) diluted 1:1000 in PBS for 1 h at room temperature. After three washing steps with PBS for 5 min, cryosections were covered with a DAKO fluorescent mounting medium (Agilent, Santa Clara, CA, USA). For laminin-332 staining, slides were incubated for 1.5 h with a human-specific anti-laminin alpha 3/laminin-5 antibody (#MAB2144; R&D systems, Minneapolis, MN, USA) diluted 1:2000 in 1× blocking reagent (Roche Diagnostics GmbH, Mannheim, Germany) in TBS-TX. Staining with secondary antibody and DAPI was performed in the same way as for C17. Cryosections were analyzed using the confocal laser scanning microscope Axio Observer Z1 attached to LSM700 (Carl Zeiss).

### 4.9. H&E Staining of Skin Equivalents

Paraffin sections of 8 µm were used for hematoxylin and eosin stainings, where hematoxylin stains the cell nuclei and eosin stains the extracellular matrix and cytoplasm. After 30 washing steps with H_2_O, slides were re-incubated for 6 min in Mayer’s hemalum solution (Merck, Kenilworth, IL, USA) followed by another 30 washing steps in H_2_O. The cryosections are dipped 10 times in a 0.3% HCl/EtOH solution, 30 times in H_2_O and further incubated for 2 min in a 0.5% Eosin G solution (Merck, Kenilworth, IL, USA). Slides were then washed 30 times in H_2_O, 30 times in isopropanol, and 30 times in HistoChoice^®^ Clearing Agent (Merck, Kenilworth, IL, USA). Finally, paraffin sections were covered with ROTI^®^Histokitt (Carl Roth, Karlsruhe, Germany) and stored at 4 °C.

## Data Availability

Not applicable.

## Supplementary Materials

The following supporting information can be downloaded at: https://www.mdpi.com/article/10.3390/ijms24065197/s1.
