# Supplementary material for: A Novel Fluorescence-Based Screen of Gene Editing Molecules for Junctional Epidermolysis Bullosa
Source: Int J Mol Sci. 2023 Mar 8;24(6):5197. doi: 10.3390/ijms24065197 (PMC10049061; doi:10.3390/ijms24065197)
Supplement: Supplementary file 1 [file ijms-24-05197-s001.zip › ijms-2236162-supplementary.pdf]

Supplemental Information

A novel fluorescence-based screen of gene editing molecules for junctional epidermolysis bullosa

Janine Zwicklhuber, Thomas Kocher, Bernadette Liemberger, Stefan Hainzl, Johannes Bischof, Dirk Strunk, Anna M. Raninger, Iris Gratz, Verena Wally, Christina Guttmann-Gruber, Josefina Piñón Hofbauer, Johann W. Bauer and Ulrich Koller

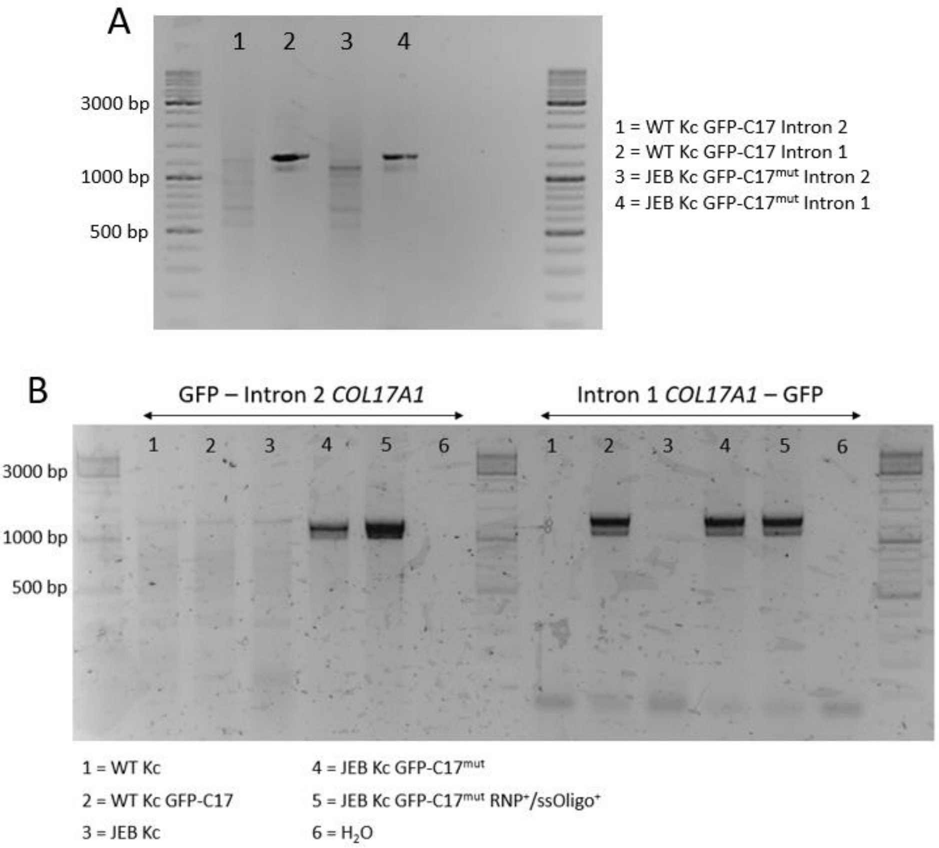

Figure S1. Integration PCRs of WT and JEB GFP-C17 and RNP/ssOligo-treated of GFP-C17^{mut}-expressing JEB cells. A) Primers specific for 1&3) GFP - Intron 2 *COL17A1* and 2&4) Intron 1 of *COL17A1* - GFP were used to amplify the target regions. Gel electrophoresis confirmed the genomic integration of the donor template in both WT and JEB Kc expressing GFP-C17. **B)** Gel electrophoresis of RNP/ssOligo-treated GFP-C17^{mut}-expressing JEB cells confirmed the genomic integration of the donor template. WT and JEB patient keratinocytes were used as control.

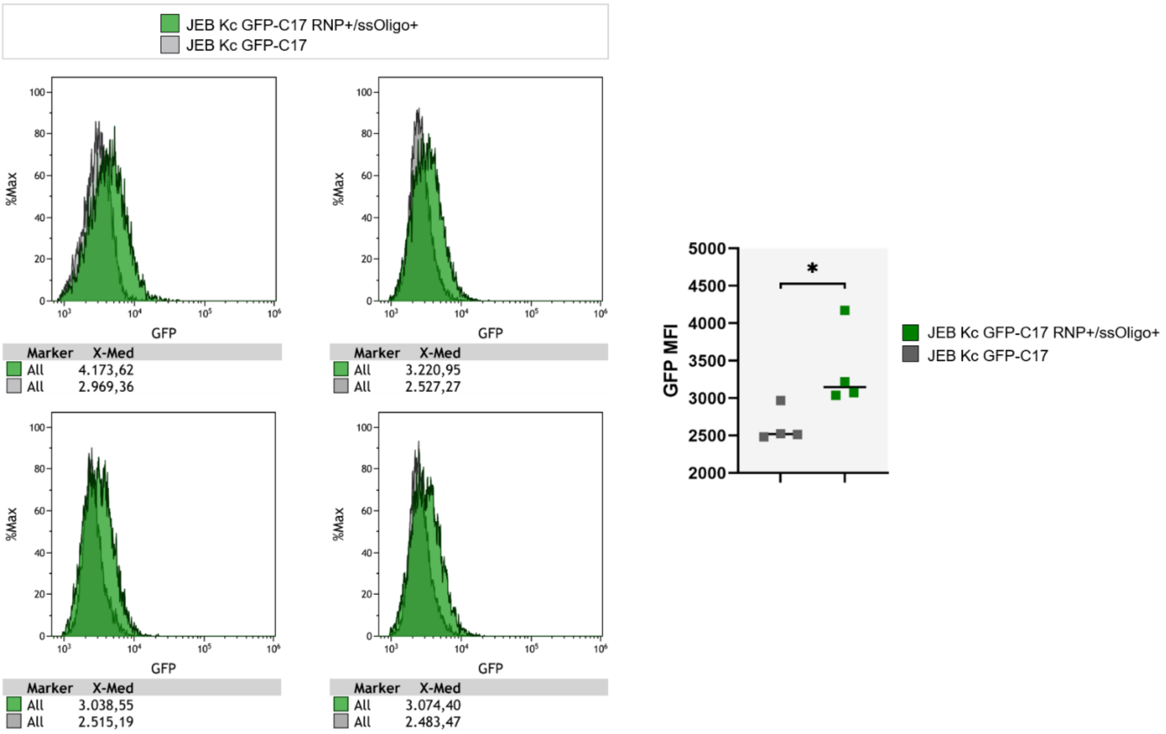

Figure S2. CRISPR/Cas9-mediated GFP-C17 restoration in JEB keratinocytes.

Flow cytometric analyses of RNP$^+$/ssODN$^+$-treated (dark green) and untreated (ligray) GFP-C17 JEB cells, showing a significant increase in GFP median fluorescence intensity (MFI, denoted by X-Med in histograms) upon gene repair in 4 individual experiments. Statistical analysis (paired Student's t test) was performed using GraphPad Prism 9.

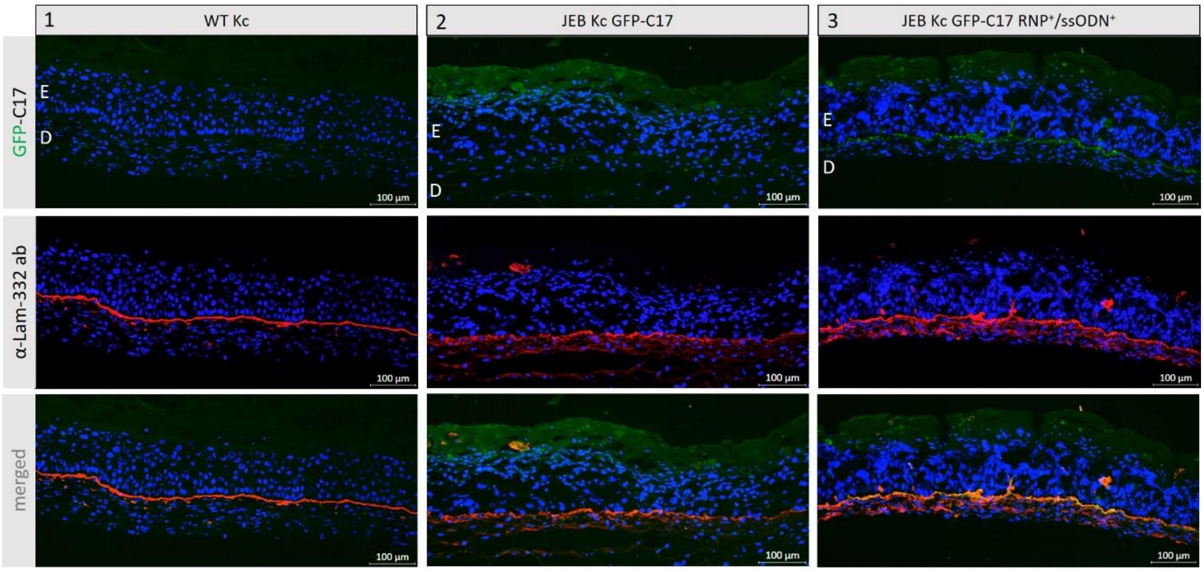

Figure S3. Laminin-332 IF stainings of JEB-derived skin equivalents after RNP/ssODN-treatment. Immunofluorescence staining performed on cryosections showed an accurate laminin-332 expression (red fluorescence) in the BMZ of SEs derived from WT keratinocytes (1). SEs from untreated GFP-C17^{mut}-expressing JEB

Kc showed fewer laminin-332 and no visible GFP-C17 along the BMZ (2), whereas immunofluorescence staining of 3D SEs expanded from RNP/ssODN-treated JEB cells revealed accurate colocalization of GFP-C17 (green fluorescence) with laminin-332 (red fluorescence) within the BMZ (3). Cell nuclei were stained with 4', 6-Diamidino-2-phenylindol (DAPI, blue). E= Epidermis ; D= Dermis.
